# BMI as a risk factor for the development of chronic rhinosinusitis: a prospective population-based study

**DOI:** 10.1007/s00405-022-07320-y

**Published:** 2022-03-19

**Authors:** Ulrika K. E. Clarhed, Linus Schiöler, Kjell Torén, Anne Kristin M. Fell, Johan Hellgren

**Affiliations:** 1 Department of Otorhinolaryngology, Head and Neck Surgery, Institute of Clinical Sciences, Sahlgrenska Academy, University of Gothenburg, Gröna Stråket 9, 413 45 Göteborg, Sweden; 2 Dept of Otorhinolaryngology, Region Västra Götaland, Sahlgrenska University Hospital, Göteborg, Sweden; 3 Department of Occupational and Environmental Medicine, Telemark Hospital, Skien, Norway; 4 Department of Community Medicine and Global Health, Institute of Health and Society, University of Oslo, Oslo, Norway; 5 Occupational and Environmental Medicine, Institute of Medicine, Sahlgrenska Academy, University of Gothenburg, Göteborg, Sweden

**Keywords:** Body mass index, Sinusitis, Inflammation, Obesity, Population

## Abstract

**Purpose:**

Obesity is a growing, global health problem and previous cross-sectional studies have demonstrated an association between obesity and chronic rhinosinusitis (CRS). There is, however, a lack of prospective studies regarding the impact of obesity on developing (new-onset) CRS.

**Methods:**

Questionnaire-based data (*n* = 5769) relating to new-onset CRS and Body Mass Index (BMI) were collected in 2013 and 2018 from the Telemark population study in Telemark, Norway. Odds ratios for the risk of new-onset CRS in 2018 in relation to BMI in 2013 were calculated, adjusted for smoking habits, asthma, gender and age.

**Results:**

When comparing the group with normal weight (18.5 ≤ BMI < 25) with the obese group (BMI ≥ 30), the odds of new-onset CRS was 53% higher [OR 1.53 (1.11, 2.10)] in the obese group.

**Conclusion:**

CRS is a multifactorial disease with different phenotypes and it is important to consider obesity when assessing patients with CRS in a clinical setting.

## Introduction

Obesity is a common health concern worldwide [1]. Obesity is most commonly considered to be caused by excess energy consumption (dietary intake) relative to energy expenditure (energy loss via metabolic and physical activity). However, the aetiology behind obesity is more complex, with the interaction of genetic, environmental, social and economic factors [2].

Obesity has been identified as an important risk factor for asthma [3, 4]. The suggested mechanism is that obesity causes a local and systemic subclinical inflammation which appears to contribute to airway inflammation, decrease in lung function and asthma exacerbation [5].

Chronic rhinosinusitis (CRS) is an inflammation of the nose and the paranasal sinuses [6] that affects around 11% of the population in Europe [7, 8] and has substantial effects on health-related quality of life [9, 10]. There is a well-known association between asthma and CRS [11–13]. Smoking is also an important risk factor for CRS [6]. Factors linking the mucosal inflammation in CRS and asthma could be related to obesity and its potential to contribute to airway inflammation. Previous cross-sectional studies have demonstrated an association between obesity and CRS [14–16]. Nam et al. investigated 32,384 individuals aged 19–86 years and, after performing a cross-sectional analysis, they found that the prevalence of CRS with nasal polyps was higher in subjects with obesity [16]. Bhattacharyya conducted a cross-sectional analysis of a medical panel survey in the United States and found an adjusted odds ratio of 1.31 (1.18, 1.45) for CRS when obesity was present [14]. Kim et al. found that 184 adult patients with CRS undergoing endoscopic sinus surgery had a significantly higher mean body mass index (BMI) (24.7 kg/m^2^) compared with controls (23.2 kg/m^2^) [15]. The percentage of obese patients (defined as BMI > 25 kg/m^2^) was also higher in the CRS group than in controls, 42.9% as compared to 24.3%.

To our knowledge, there are no previous studies investigating BMI and its possible association with the development of CRS in a prospective study design. In this study, we have investigated the impact of BMI on new-onset CRS during a five-year observation period in a large population-based cohort from the Telemark region in southern Norway.

## Materials and methods

### Study population and design

The Telemark Study is a prospective study of a random general population cohort from the County of Telemark, Norway (population of about 170,000). In 2013, a random sample of 50,000 subjects of working age, 16–50, was drawn from the national Norwegian population registry. A postal questionnaire including questions assessing CRS, asthma, smoking, body height and weight was sent out. A 5-year follow-up of the cohort was made in 2018 using the same questionnaire. A more comprehensive description of the study can be found elsewhere [7, 17].

**Fig. 1:**
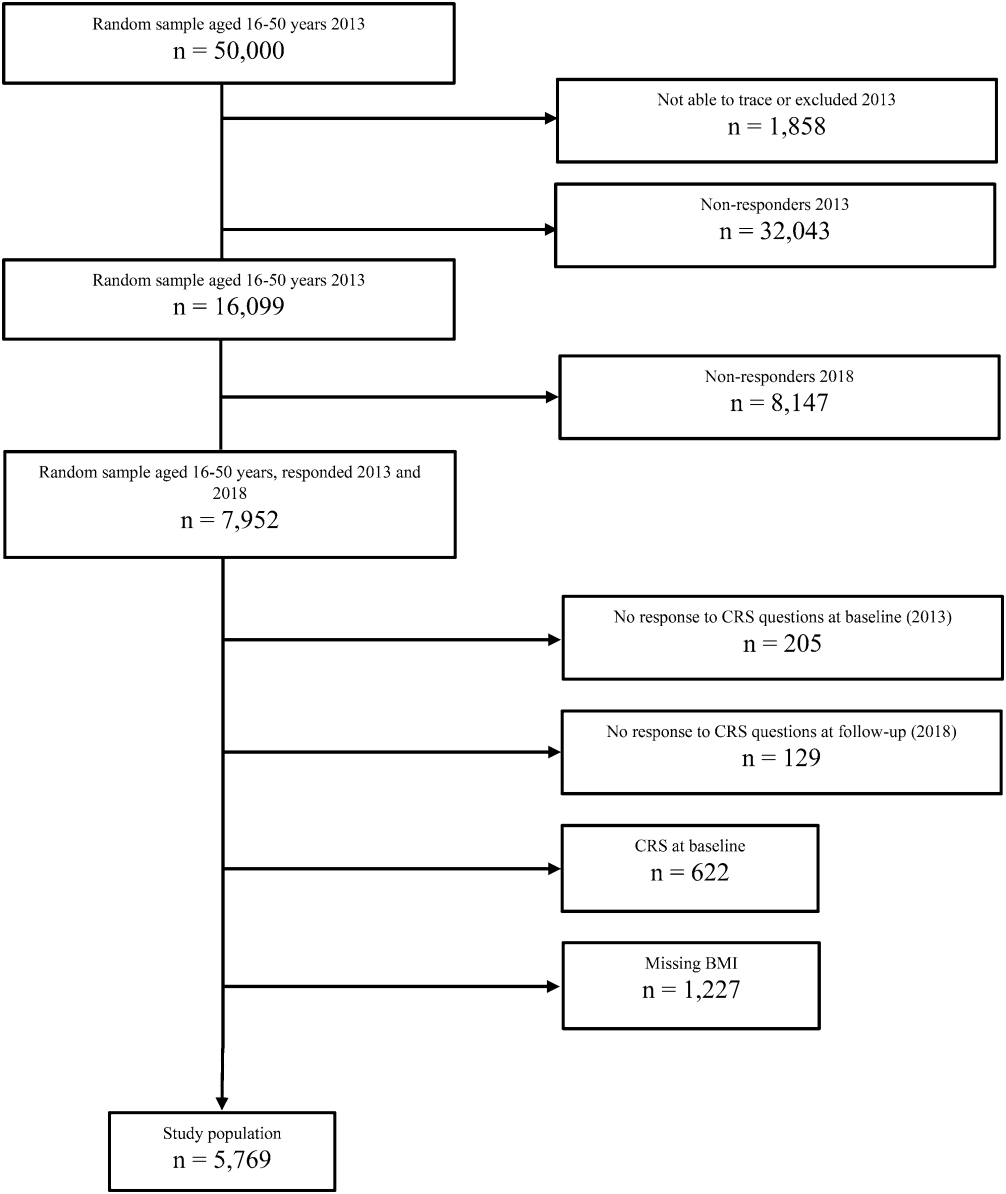
Flow chart of the study population

A total of 7952 subjects answered the questionnaire in both 2013 and 2018. Since the present study was aimed at new-onset CRS in relation to BMI, subjects reporting CRS at baseline in 2013 were excluded from the analyses. Subjects who did not answer the CRS questions in 2013 or 2018 were also excluded, as well as 1227 subjects with missing answers on either height or weight where BMI could not be calculated. A flow chart of the study population is shown in Fig. 1.

CRS was defined according to the European position paper on rhinosinusitis and nasal polyps (EPOS) criteria, as inflammation of the nose and the paranasal sinuses characterised by the presence of two or more symptoms for ≥ 12 weeks, of which one should be either nasal blockage/obstruction/congestion or nasal discharge (anterior/posterior nasal drip), called “major symptoms”, as well as additional symptoms, such as facial pain/pressure and/or a reduction in or the loss of smell, called “minor symptoms” [6].

**Fig. 2:**
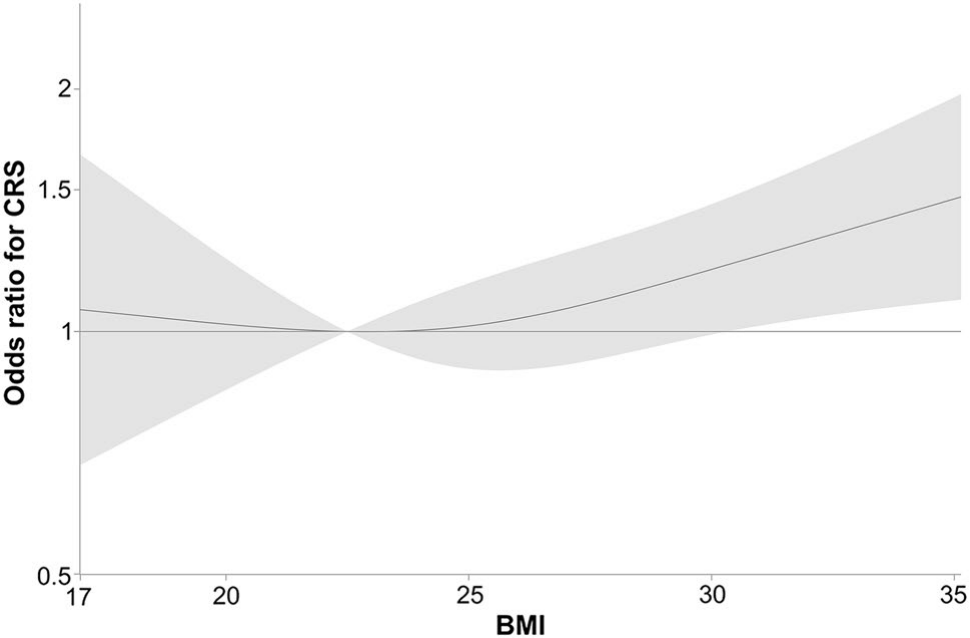
Odds ratio (OR) for new-onset CRS in relation to BMI (multivariable logistic regression analysis of risk of new-onset CRS and BMI, adjusted for gender, smoking, asthma and age). The grey area represents the 95% confidence intervals

BMI was calculated on the basis of self-reported weight and height and categorised in accordance with the guidelines from Centres for Disease Control and Prevention (CDC) [18]. The World Health Organisation (WHO) uses the same categorisation and classifications apart from overweight, which is classified as pre-obesity according to the WHO [19]. BMI was used both as a continuous variable (Fig. 2) and divided into the CDC group guidelines with the following cut-off limits: < 18.5 kg/m^2^ underweight, 18.5 to < 25 kg/m^2^ normal weight, 25.0 to < 30 kg/m^2^ overweight and ≥ 30 kg/m^2^ obesity (Table 2). For subjects with missing height but observed weight at baseline, height at follow-up was used to calculate BMI.

Smoking was assessed using the following questions; ‘Do you smoke every day*?*’, ‘Do you smoke only occasionally*?’* and *‘*Did you use to smoke*?’*. ‘Ever smoking*’* was defined as giving a positive answer to any of the three questions. ‘Current smoking*’* was defined as a positive answer to either ‘Do you smoke every day*?*’ or ‘Do you only smoke occasionally*?*’. Asthma was defined as a positive answer to the question ‘Has a physician ever diagnosed you with asthma?’.

### Statistical analysis

Odds ratios were estimated using multivariable logistic regression. Restricted cubic splines were used to model continuous variables: for age, we placed knots at the 10th, 50th and 90th percentiles [20], corresponding to 23, 41 and 49. For BMI, we placed knots at 18.5, 25 and 30, corresponding to the CDC categories. Data on BMI were missing in 1227 subjects and they were therefore excluded. A small number (*n* = 92) of subjects had missing data on asthma or smoking status and were excluded from the regression analyses.

All the analyses were performed using SAS Version 9.4M6 (SAS Institute, Cary, NC). P values of < 0.05 were considered statistically significant.

## Results

The descriptive statistics of the study population are presented in Table 1. One per cent of the study population (*n* = 5769) was underweight, 50% had normal weight, 35% were overweight and 14% were obese.

Odds ratios (OR) from the multivariable logistic regression analysis of risk of new-onset CRS in 2018 in relation to BMI in 2013, adjusted for gender, smoking habits, asthma and age, can be seen in Table 2. When comparing the group with normal weight with the obese group, the odds of new-onset CRS was 53% higher [OR 1.53 (1.11, 2.10)] in the obese group.

**Table 1:** Description of study population (*n* = 5769) at baseline (2013) with regard to age, gender, smoking habits and asthma

	BMI				
	Underweight BMI < 18.5 (*n* = 83)	Normal weight 18.5 ≤ BMI < 25 (*n* = 2886)	Overweight 25 ≤ BMI < 30 (*n* = 2001)	Obesity BMI ≥ 30 (*n* = 799)	Total (*n* = 5769)
Age (years)					
Median (interquartile range)	28 (20–40)	38 (27–45)	42 (35–47)	43 (37–47)	40 (31–46)
Gender *n* (%)					
Female	66 (2)	1970 (58)	929 (28)	410 (12)	3375 (100)
Male	17 (1)	916 (38)	1072 (45)	389 (16)	2394 (100)
Smoking *n* (%)					
Never	55 (2)	1794 (54)	1074 (32)	395 (12)	3318 (100)
Past	6 (0)	531 (43)	497 (40)	215 (17)	1249 (100)
Current^a^	22 (2)	552 (47)	425 (36)	186 (16)	1185 (100)
Asthma *n* (%)					
No	73 (1)	2612 (51)	1792 (35)	693 (13)	5170 (100)
Yes	7 (1)	225 (43)	189 (36)	101 (19)	522 (100)

^a^Including occasional smokers

**Table 2:** Odds ratio (OR)

Study population	No. CRS (*n*)	CRS (*n*)	OR (95% CI)
Underweight	75	5	1.20 (0.50, 2.93)
Normal weight	2682	147	1 (ref)
Overweight	1882	95	0.93 (0.71, 1.23)
Obese	730	61	**1.53 (1.11, 2.10)**

Statistically significant results are marked in bold

Multivariable logistic regression analysis of new-onset CRS in 2018 in relation to BMI in 2013 adjusted for gender, smoking habits, asthma and age. The data regarding asthma or smoking were missing in 92 subjects and they were therefore not included. Underweight = BMI < 18.5, normal weight = 18.5 ≤ BMI < 25, overweight = 25 ≤ BMI < 30, obese = BMI ≥ 30

The characteristics of the subjects with a missing BMI are shown in Table 3, when compared with the study population. A similar distribution of gender, asthma, smoking and new-onset CRS can be seen in both groups.

**Table 3:** Characteristics of subjects with missing BMI compared with study population

	Study population (*n* = 5769)	Missing BMI (*n* = 1,227)
Gender % (*n*)		
Female	59 (3375)	61 (748)
Male	41 (2394)	39 (479)
Smoking % (n)		
Never smoking	58 (3318)	56 (682)
Past smoking	22 (1249)	26 (313)
Current smoking	21 (1185)	19 (230)
Asthma % (*n*)		
Yes	9 (522)	10 (127)
No	91 (5170)	90 (1085)
New-onset CRS 2013–2018% (*n*)		
Yes	5 (313)	6 (76)
No	95 (5456)	94 (1151)

Figure 2 presents OR for new-onset CRS in relation to BMI with multivariable logistic regression analysis. The variables were adjusted for gender, smoking, asthma and age. When BMI was used as a continuous variable, the risk of new-onset CRS increased with a higher BMI.

## Discussion

In this prospective study of a large random population cohort, a higher BMI was associated with an increased risk of developing CRS during a five-year observation period. This association was present both when analysing BMI as a continuous variable (Fig. 2) and when categorising BMI according to CDC definitions and comparing normal weight (18.5 ≤ BMI < 25) with obesity (BMI ≤ 30) (OR 1.53 (1.11–2.10) (Table 2). To our knowledge, this study is the first to confirm BMI as a risk factor for the development of CRS in a prospective design. As patients with CRS have chronic disease where causal treatment is lacking and different phenotypes and endotypes exist, BMI should be taken into consideration when CRS patients are at the clinic [6].

One major strength of this study is the prospective design. By excluding all subjects reporting CRS according to the EPOS criteria, at baseline, we have only studied new-onset CRS during the five-year observation period. Our results show that obesity is a risk factor for the development of CRS, even when other known risk factors such as asthma and smoking are taken into consideration. This confirms previous cross-sectional studies indicating the existence of an association between CRS and BMI. In a large cross-sectional study from 2013, Bhattacharyya et al. found an increased prevalence of obesity in patients suffering from CRS [14]. Another cross-sectional study from 2016 found that obesity was a risk factor for CRS with nasal polyps, but its data did not support a connection between a higher BMI and CRS without nasal polyps [21]. Obesity was defined as a BMI of ≥ 25, whereas in our study we define obesity as a BMI of ≥ 30, in accordance with CDC [18]. Lee et al. found that CRS was more prevalent in individuals with the metabolic syndrome (a high triglyceride level, a reduced high-density lipoprotein level and elevated blood pressure) than in individuals without the metabolic syndrome [22]. Sidell et al. found no association between obesity and CRS in schoolchildren [23]. This study included children aged 6–17 years, whereas our study investigates adults aged 16–50 years, which could be a reason for the difference in results. Kanagalingam et al. found that obesity did not affect the severity of sinonasal disease in asthma [24]. That study used a validated questionnaire [25] to assess sinonasal disease severity, whereas in our study we use the EPOS criteria [6] to define chronic rhinosinusitis, which could explain the discrepancy in the results.

Obesity itself appears to cause a metabolic inflammatory state, defined as a low-grade, chronic inflammation orchestrated by metabolic cells and inflammatory markers in response to excess nutrients and energy [26]. Previous studies have highlighted obesity as an important risk factor for asthma, but the mechanisms behind this relationship still remain elusive [27, 28]. The connection between CRS and lower respiratory airway disease, such as asthma, is well known and was also taken into account in the analyses in this study [11–13]. The inflammatory response caused by obesity appears to contribute to airway inflammation, decrease in lung function and asthma exacerbation [5]. Obesity in patients with asthma also results in a higher symptom burden, as well as poorer asthma control and a higher consumption of asthma medication, which could also be a factor in CRS, but this needs to be studied in more detail [29].

The Telemark Study is a large prospective population-based cohort designed to study airway inflammatory disease and risk factors such as CRS. One of the main advantages of the population-based design is that it enables the inclusion of subjects who have CRS symptoms but who have not been in contact with health care, in contrast to other study designs based on the hospital cohorts of CRS patients. EPOS has a special symptom-based definition of CRS for the purpose of questionnaire-based epidemiological research. In the absence of a nasal inspection, it is however not possible to exclude other causes of the sinonasal symptoms or to differentiate between nasal polyps and other CRS. A strength of the study is that the questions used in the Telemark Study have been validated and frequently used before [30–32].

When conducting epidemiological research, it is important to consider recall bias and how it might affect the outcome. There is a risk that individuals with CRS are more prone to recall their symptoms than their counterparts who did not develop CRS; thus, resulting in a skewed distribution of the investigated symptoms or characteristics. The studied variables, such as smoking, asthma and sinonasal symptoms during the past 12 weeks, are probably less likely to be over- or underestimated and the results show that their prevalence was as expected. This may not, however, apply to BMI. Previous studies illustrate that individuals have a tendency to overestimate their height and underestimate their weight, resulting in a falsely low BMI [33]. This tendency is greater in individuals who are overweight or obese compared with individuals of normal weight. However, in this study, the application of this theory would mean that the individuals who reported being overweight or obese have a higher BMI in reality, thus resulting in a stronger correlation between CRS and obesity. Between the Nordic countries, the estimated prevalence of overweight and obesity is similar in Norway, Sweden, Denmark and Finland [34]. In the general population in Norway in 2019, the distribution of self-reported BMI was 2% underweight, 35% overweight and 16% obese. Forty-seven per cent had a normal BMI [35]. This is similar to our results of 1%, 35%, 14% and 50% (Table 1).

1,227 subjects failed to answer the questions regarding weight and height and were therefore not eligible. However, the analyses of these subjects showed that the overall five-year incidence of CRS and the distribution of smoking habits, asthma and gender were in accordance with the study population (Table 3).

Low vitamin D status has been associated with obesity [36, 37] and a recent meta-analysis by Li et al. illustrated an association between lower serum vitamin D status and CRS [38]. Vitamin D supplementation has also been shown to prevent acute respiratory infections [39]. The Telemark study did not include any data on serum vitamin D, however the role of vitamin D in regard to CRS and obesity is interesting and warrants further research.

Obesity has also been linked to sleep disturbances, in particular obstructive sleep apnea which in itself is connected with numerous chronic health conditions [40]. Prolonged sleep deficiency can lead to a chronic, systemic low-grade inflammation associated with various diseases that have an inflammatory component, such as diabetes [41]. There are data suggesting a correlation between obstructive sleep apnea and asthma [42]. Sleep disturbances and its concomitant inflammatory component may also be associated with CRS, however, this connection needs to be studied further. Finally, there is a connection between obesity and diabetes mellitus [43] and there is also data that show that subjects with diabetes mellitus and CRS who underwent functional endoscopic sinus surgery (FESS) report worse quality of life after surgery than those without diabetes mellitus [44].

As outlined by the EPOS, it is important to study different phenotypes of CRS [6]. Obesity is a sign of underlying disease and/or the interaction of genetic, environmental, social and economic factors that can facilitate the development of CRS and asthma.

## Conclusion

In this prospective study of BMI as a risk factor for CRS, the risk of new-onset CRS increased with a higher BMI. Our results show that obese individuals ran a 53% increased risk of acquiring new-onset CRS [OR 1.53 (CI 1.11, 2.10)] when compared with individuals of normal weight. BMI should be taken into account when assessing CRS in a clinical setting.

## References

[1] Arroyo-Johnson C, Mincey KD (2016). Obesity epidemiology worldwide. Gastroenterol Clin North Am.

[2] Wright SM, Aronne LJ (2012). Causes of obesity. Abdom Imaging.

[3] Peters U, Dixon AE, Forno E (2018). Obesity and asthma. J Allergy Clin Immunol.

[4] van der Plaat DA (2020). Mendelian randomisation supports causal link between obesity and asthma. Thorax.

[5] Miethe S, Karsonova A, Karaulov A, Renz H (2020). Obesity and asthma. J Allergy Clin Immunol.

[6] Fokkens WJ, Lund VJ, Hopkins C (2020). European position paper on rhinosinusitis and nasal polyps 2020. Rhinology.

[7] Clarhed UKE, Svendsen M, Schiöler L (2018). Chronic rhinosinusitis related to occupational exposure: the telemark population study. J Occup Environ Med.

[8] Hastan D, Fokkens WJ, Bachert C (2011). Chronic rhinosinusitis in Europe–an underestimated disease. A GA2LEN study. Allergy.

[9] Rudmik L, Smith TL (2011). Quality of life in patients with chronic rhinosinusitis. Curr Allergy Asthma Rep.

[10] Ylitalo-Heikkilä M, Virkkula P, Sintonen H, Lundberg M, Roine RP, Hytönen M (2018). Different rhinologic diseases cause a similar multidimensional decrease in generic health-related quality of life. Clin Otolaryngol.

[11] Ostovar A, Fokkens WJ, Vahdat K, Raeisi A, Mallahzadeh A, Farrokhi S (2019). Epidemiology of chronic rhinosinusitis in Bushehr, southwestern region of Iran: a GA2LEN study. Rhinology.

[12] Philpott CM, Erskine S, Hopkins C (2018). Prevalence of asthma, aspirin sensitivity and allergy in chronic rhinosinusitis: data from the UK National Chronic Rhinosinusitis Epidemiology Study. Respir Res.

[13] Tomisa G, Horváth A, Szalai Z, Müller V, Tamási L (2019). Prevalence and impact of risk factors for poor asthma outcomes in a large, specialist-managed patient cohort: a real-life study. J Asthma Allergy.

[14] Bhattacharyya N (2013). Associations between obesity and inflammatory sinonasal disorders. Laryngoscope.

[15] Kim TH, Kang HM, Oh IH, Yeo SG (2015). Relationship between otorhinolaryngologic diseases and obesity. Clin Exp Otorhinolaryngol.

[16] Nam JS, Roh YH, Fahad WA (2021). Association between obesity and chronic rhinosinusitis with nasal polyps: a national population-based study. BMJ Open.

[17] Abrahamsen R, Svendsen MV, Henneberger PK (2016). Non-response in a cross-sectional study of respiratory health in Norway. BMJ Open.

[18] Centres for Disease Control and Prevention (2021) Body mass index (BMI). https://www.cdc.gov/obesity/adult/defining.html Accessed 17 November 2021

[19] World Health Organization (2021) Body mass index (BMI). https://www.euro.who.int/en/health-topics/disease-prevention/nutrition/a-healthy-lifestyle/body-mass-index-bmi Accessed 17 November 2021

[20] Harrell FE (2015). Regression modeling strategies: with applications, to linear models, logistic and ordinal regression, and survival analysis.

[21] Ahn JC, Kim JW, Lee CH, Rhee CS (2016). Prevalence and risk factors of chronic rhinosinusitus, allergic rhinitis, and nasal septal deviation: results of the Korean National Health and Nutrition Survey 2008–2012. JAMA Otolaryngol Head Neck Surg.

[22] Lee EJ, Hwang HJ, Jung CM, Kim MK, Kang MS, Kim KS (2017). The relationship between chronic rhinosinusitis and metabolic syndrome. Am J Rhinol Allergy.

[23] Sidell D, Shapiro NL, Bhattacharyya N (2013). Obesity and the risk of chronic rhinosinusitis, allergic rhinitis, and acute otitis media in school-age children. Laryngoscope.

[24] Kanagalingam S, Shehab SS, Kaminsky DA, Wise RA, Lang JE, Dixon AE (2018). Effect of obesity on sinonasal disease in asthma. J Asthma.

[25] Dixon AE, Sugar EA, Zinreich SJ (2009). Criteria to screen for chronic sinonasal disease. Chest.

[26] Gregor MF, Hotamisligil GS (2011). Inflammatory mechanisms in obesity. Annu Rev Immunol.

[27] Mancuso P (2010). Obesity and lung inflammation. J Appl Physiol (1985).

[28] Shore SA (2010). Obesity, airway hyperresponsiveness, and inflammation. J Appl Physiol (1985).

[29] Klepaker G, Svendsen MV, Hertel JK (2019). Influence of obesity on work ability, respiratory symptoms, and lung function in adults with asthma. Respiration.

[30] The European Community Respiratory Health Survey II (2002) Eur Respir J 20:1071–9 10.1183/09031936.02.00046802 12449157

[31] Torén K, Brisman J, Järvholm B (1993). Asthma and asthma-like symptoms in adults assessed by questionnaires. A literature review. Chest.

[32] Torén K, Ekerljung L, Kim JL (2011). Adult-onset asthma in west Sweden–incidence, sex differences and impact of occupational exposures. Respir Med.

[33] Maukonen M, Männistö S, Tolonen H (2018). A comparison of measured versus self-reported anthropometrics for assessing obesity in adults: a literature review. Scand J Public Health.

[34] Stockmarr A, Hejgaard T, Matthiessen J (2016). Obesity prevention in the nordic countries. Curr Obes Rep.

[35] Statistisk sentralbyrå Norway (2021). https://www.ssb.no/statbank/table/06181/tableViewLayout1/ Accessed 17 November 2021

[36] Karampela I, Sakelliou A, Vallianou N, Christodoulatos G-S, Magkos F, Dalamaga M (2021). Vitamin D and obesity: current evidence and controversies. Curr Obes Rep.

[37] Saneei P, Salehi-Abargouei A, Esmaillzadeh A (2013). Serum 25-hydroxy vitamin D levels in relation to body mass index: a systematic review and meta-analysis. Obes Rev.

[38] Li B, Wang M, Zhou L, Wen Q, Zou J (2021). Association between serum vitamin D and chronic rhinosinusitis: a meta-analysis. Braz J Otorhinolaryngol.

[39] Martineau AR, Jolliffe DA, Greenberg L (2019). Vitamin D supplementation to prevent acute respiratory infections: individual participant data meta-analysis. Health Technol Assess.

[40] Muscogiuri G, Barrea L, Annunziata G (2019). Obesity and sleep disturbance: the chicken or the egg?. Crit Rev Food Sci Nutr.

[41] Besedovsky L, Lange T, Haack M (2019). The sleep-immune crosstalk in health and disease. Physiol Rev.

[42] Davies SE, Bishopp A, Wharton S, Turner AM, Mansur AH (2019). The association between asthma and obstructive sleep apnea (OSA): a systematic review. J Asthma.

[43] Chobot A, Gorowska-Kowolik K, Sokolowska M, Jarosz-Chobot P (2018). Obesity and diabetes—not only a simple link between two epidemics. Diabetes Metab Res Rev.

[44] Zhang Z, Adappa ND, Lautenbach E (2014). The effect of diabetes mellitus on chronic rhinosinusitis and sinus surgery outcome. Int Forum Allergy Rhinol.

